# Evolving trends in oral cancer burden in Europe: a systematic review

**DOI:** 10.3389/fonc.2024.1444326

**Published:** 2024-10-18

**Authors:** Amr Sayed Ghanem, Hafsa Aijaz Memon, Attila Csaba Nagy

**Affiliations:** Department of Health Informatics, Institute of Health Sciences, Faculty of Health Sciences, University of Debrecen, Debrecen, Hungary

**Keywords:** oral squamous cell carcinoma, epidemiology, GLOBOCAN, risk factors, HPV, incidence, mortality

## Abstract

**Introduction:**

Oral cavity cancer (OCC), primarily oral squamous cell carcinoma (OSCC), is a growing concern in Europe, particularly among younger populations. Preventable lifestyle factors and social determinants of health contribute significantly to the disease burden. Limited access to healthcare and delayed diagnoses further complicate treatment and reduce survival rates.

**Methods:**

This systematic literature review adhered to PRISMA guidelines to explore trends in OSCC epidemiology, etiology, diagnosis, treatment, and survival across Europe. A comprehensive search strategy using PubMed, GLOBOCAN data, and the EUROCARE-5 study identified relevant articles focusing on human populations in Europe with a primary interest in OSCC epidemiology. Only peer-reviewed publications in English with full-text access were included.

**Results:**

This study investigated the burden of OSCC across Europe, revealing variations in incidence, mortality, and prognosis. Eastern and Central Europe displayed the highest burden. Males exhibited a significantly higher risk compared to females. Age-related disparities existed in life expectancy and time to achieve favorable outcomes. HPV emerged as a growing risk factor for oropharyngeal cancer. Public health strategies should target modifiable risk factors and improve early detection.

**Conclusion:**

This review reveals concerning disparities in European OSCC. Region, sex, and age all influence burden and prognosis. Future research should focus on controlling risk factors and personalized medicine to optimize treatment. This will lead to a Europe with reduced OSCC incidence and demonstrably better patient outcomes.

## 1 Introduction

Oral cancer, specifically oral squamous cell carcinoma (OSCC), poses a significant challenge within the European healthcare system. OSCC accounts for roughly 90% of all oral malignancies, frequently targeting the tongue, lips, and floor of the mouth (1). These squamous cell carcinomas originate from the oral cavity’s lining and can develop into aggressive tumors if not detected and treated early. The complex etiology of OSCC presents obstacles in treatment and management, impacting individuals throughout their journey from diagnosis to recovery. Treatment complications like pain, salivary gland dysfunction, and swallowing difficulties further disrupt patient well-being (2). OSCC incidence rates, particularly among younger demographics, are a growing concern in Europe. While SCCs contribute significantly to new cancer diagnoses annually, preventable lifestyle factors like tobacco use, alcohol consumption, and diet along with viral infections and environmental exposures play a substantial role (3). These factors disproportionately affect socioeconomically disadvantaged populations and highlight the need for inclusive preventive measures (4). Gender disparities are also evident, with males experiencing higher rates partly due to lifestyle behaviors and less frequent dental visits. This disparity can be attributed to several factors. Males are more prone to engage in high-risk behaviors, such as tobacco and alcohol use, with a higher prevalence of heavy smoking and binge drinking, which are significant risk factors for oral cancer. Occupational exposure to carcinogens is also more frequent in industries predominantly staffed by men (5). Furthermore, studies have shown that men are generally less likely to participate in cancer screening programs compared to women, leading to later-stage diagnoses and a higher incidence of the disease (6). Additionally, many men tend to avoid seeking medical advice or delay discussing critical health issues during consultations, resulting in delayed diagnosis and less effective treatment (7). In contrast, women typically demonstrate more positive attitudes towards healthcare, including greater adherence to dental and medical services, better self-care practices, and higher levels of oral health literacy. This is reflected in the slightly higher mean and median ages at diagnosis for women, suggesting that their proactive health-seeking behaviors and earlier detection contribute to the observed gender differences in oral cancer incidence (8).

Limited access to healthcare and delayed screenings contribute to late-stage diagnoses, significantly reducing the chances of successful treatment (9). Early detection through dentist-led screening programs is crucial for improving survival rates. Early interventions like surgery or radiotherapy have proven effective, emphasizing the need for enhanced dental professional education to bolster detection and prevention efforts. Survival rates for OSCC patients vary considerably depending on the stage of diagnosis and treatment timeliness. Early detection significantly improves outcomes, while late-stage diagnoses often lead to poorer prognoses and increased mortality (10). Understanding these trends, as identified in global and European studies, underscores the importance of early detection initiatives in reducing the burden of OSCC.

This systematic review aims to provide a comprehensive overview of OSCC in Europe, encompassing its epidemiology, etiology, diagnosis, treatment, survival rates, mortality, and knowledge gaps. By analyzing trends over time, including changes in incidence, mortality, and survival, we can formulate evidence-based policies. This empirical understanding will guide policymakers and healthcare stakeholders toward implementing more effective preventive measures, screening programs, and improved treatment modalities. Ultimately, the convergence of scientific research, evidence-based analyses, and concerted policy initiatives stands poised to mitigate the impact of OSCC within Europe. By charting a course towards early detection, tailored interventions, and bolstered healthcare infrastructure, the collective effort aims not only to alleviate the burdens borne by affected individuals but also to forge a future where the prevalence of OSCC is diminished, enabling healthier societies and stronger healthcare systems across Europe.

## 2 Methodology

This review adhered to the Preferred Reporting Items for Systematic Reviews and Meta-Analyses (PRISMA) guidelines to ensure transparency and comprehensiveness. A systematic literature search was conducted on PubMed between November 2023 and April 2024. Only articles published between 2018 and 2023 were included to ensure the most recent data was considered.

### 2.1 Data sources

To gain a well-rounded understanding of OSCC epidemiology in Europe, data was utilized from a variety of sources:

#### 2.1.1 Literature search

A systematic literature search was conducted on PubMed, a bibliographic database maintained by the National Institutes of Health (NIH). The search strategy included a combination of Medical Subject Headings (MeSH) terms such as “oral squamous cell carcinoma,” “oral cavity cancer,” “epidemiology,” “Europe,” and relevant keywords encompassing various epidemiological aspects. These keywords targeted disease burden (e.g., “incidence,” “mortality,” “prevalence,” “survival”), risk factors and etiology (e.g., “tobacco use,” “alcohol consumption,” “human papillomavirus infection”), diagnosis and treatment (e.g., “diagnosis,” “screening,” “treatment”), and prognosis (e.g., “prognosis,” “life expectancy”). By incorporating this broad range of keywords, we aimed to capture a comprehensive understanding of OSCC epidemiology in the European context.

#### 2.1.2 Global cancer observatory (GLOBOCAN

Incidence and mortality data for oral squamous cell carcinoma (OSCC) across Europe were retrieved from the GLOBOCAN database, a resource curated by the International Agency for Research on Cancer (IARC). Data from the 2022 edition of GLOBOCAN were utilized to identify temporal trends.

#### 2.1.3 EUROCARE-5 study

Data on prognosis and life expectancy for patients diagnosed with oral squamous cell carcinoma and pharyngeal cancers were obtained from the EUROCARE-5 study, a large-scale population-based initiative investigating cancer survival across Europe.

### 2.2 Eligibility criteria

Articles retrieved from the PubMed search were included in the review if they met the following criteria:

#### 2.2.1 Focus

The primary focus had to be OSCC epidemiology. This includes investigations into the incidence, mortality, survival rates, prevalence, risk factors, etiology, public health interventions, diagnosis, treatment, and prognosis. These facets provided valuable insights into the broader understanding of the disease burden and trends in Europe.

#### 2.2.2 Population

The study population had to be human and specifically located in Europe.

#### 2.2.3 Study design

Observational studies (cohort studies, case-control studies, cross-sectional studies) and clinical trials with relevant epidemiological data were considered for inclusion. Peer-reviewed research articles, systematic reviews, and meta-analyses were prioritized to ensure methodological rigor and data reliability.

#### 2.2.4 Language

The language of published articles included in the current review was restricted to English. While this criterion may exclude relevant research in other languages, it was implemented to ensure the accuracy and consistency of data interpretation and analysis.

#### 2.2.5 Full-text accessibility

Only articles for which full-text access was obtainable were included in the review. This included articles freely available online or accessible through our institutional subscriptions. Paywalled articles were not pursued due to resource limitations.

### 2.3 Exclusion criteria

Articles retrieved from the PubMed search were excluded based on the following criteria:

#### 2.3.1 Duplicates

Identified duplicates were removed using Zotero, a reference management software, to ensure efficient data management and avoid redundancy in the review process.

#### 2.3.2 Non-peer-reviewed formats

Formats excluded from the review included conference abstracts, editorials, and letters lacking sufficient methodological rigor or relevance to the European context. However, to ensure a comprehensive understanding of the topic, relevant data from grey literature sources were considered. These sources included governmental or organizational reports focusing on European populations. A critical appraisal tool, the AACODS checklist (11) was used to evaluate the methodological quality of these grey literature sources. Grey literature databases, including the Global Health Observatory (GHO) and Scopus, were searched to ensure comprehensive coverage of relevant materials.

#### 2.3.3 Relevance and geographic specificity

Articles not directly addressing OSCC epidemiology or focusing on populations outside of Europe were excluded. Geographically, Europe was defined to encompass all member states of the European Union, as well as non-member states with membership in the WHO European Region.

## 3 Results

**Figure 1:**
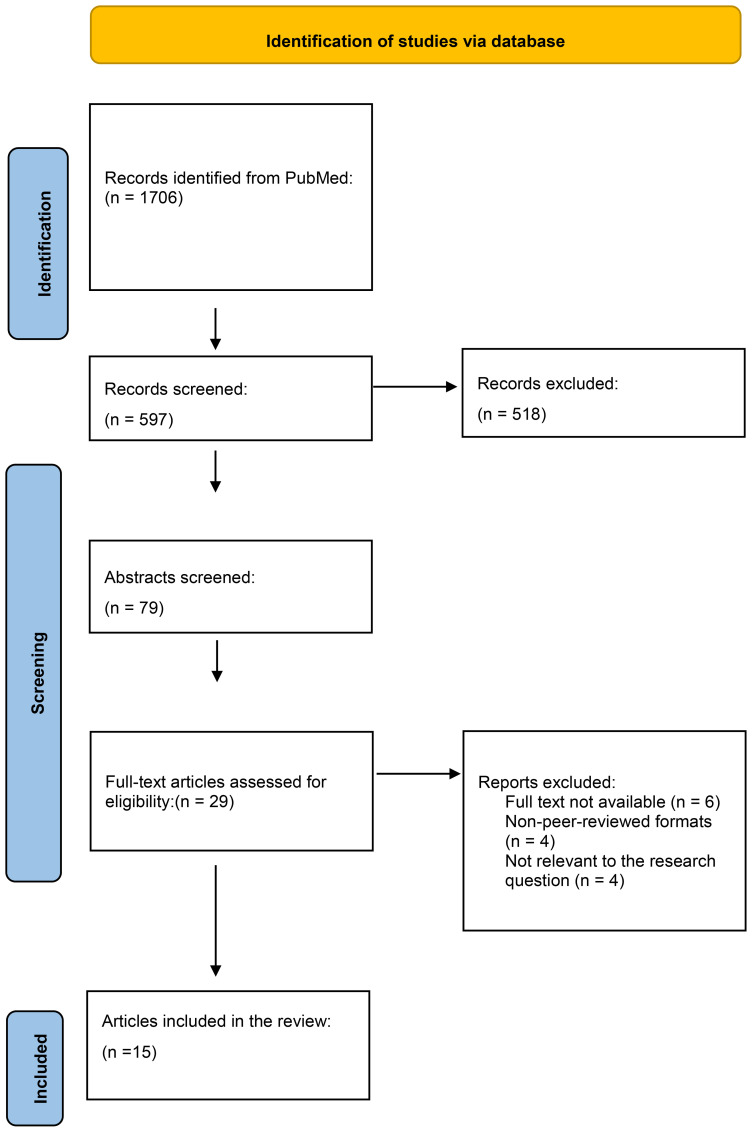
PRISMA Flow Diagram. This diagram illustrates the number of articles included and excluded at each stage of the screening and review process, leading to the selection of 15 studies for analysis in this systematic review of oral cancer.

The initial search on PubMed identified 1706 records. After removing duplicates using Zotero, a total of 597 articles were screened based on titles and abstracts. Following this screening, 518 articles were excluded. A further 79 articles underwent full-text assessment for eligibility, of which 29 were excluded. Ultimately, 15 articles were included in the review. The PRISMA flow diagram (**Figure 1**) provides a detailed breakdown of the selection process.

### 3.1 Burden of oral squamous cell carcinoma in Europe

In-depth studies conducted previously across Europe have highlighted the complex factors that influence OSCC outcomes. These comprehensive investigations have uncovered age-related variations in life expectancy, regional disparities in incidence and mortality rates, and prognostic indicators associated with recurrence. These findings have served as the foundation for developing personalized treatment strategies and post-treatment monitoring guidelines, paving the way for enhanced patient outcomes. In Europe, lip and oral cavity cancers, which includes OSCC as the most predominant subtype, remain a pressing public health concern, accounting for over 130,000 new cases and over 60,000 deaths annually. The burden of the disease varies across European regions, with Eastern and Central Europe experiencing higher incidence rates. Examining the latest GLOBOCAN 2022 data on oral cancers can provide a broader global perspective on the disease burden (12) by analyzing trends in incidence, mortality, and prevalence across different geographical regions (**Tables 1**–**3**).

**Table 1:** Estimated oral cancer incidence (all cases), both sexes, Europe (2022).

Both Sexes					Males				Females			
Cancer type	Number	ASR (World)	Crude rate	Cumulative risk	Number	ASR (World)	Crude rate	Cumulative risk	Number	ASR (World)	Crude rate	Cumulative risk
All cancers excl. non-melanoma skin cancer	4143643	265.8	553.8	26.9	2168488	299.7	599.7	30.6	1975155	243.2	510.8	24.0
Lip, oral cavity	62103	4.2	8.3	0.50	41607	6.4	11.5	0.78	20496	2.2	5.3	0.26
Salivary glands	9144	0.61	1.2	0.07	5258	0.75	1.5	0.08	3886	0.51	1.0	0.05
Oropharynx	29807	2.2	4.0	0.28	23286	3.8	6.4	0.47	6521	0.87	1.7	0.11
Nasopharynx	4518	0.39	0.60	0.04	3274	0.59	0.91	0.06	1244	0.20	0.32	0.02
Hypopharynx	16009	1.2	2.1	0.15	14097	2.2	3.9	0.28	1912	0.25	0.49	0.03
Oesophagus	53501	3.3	7.2	0.42	40508	5.7	11.2	0.72	12993	1.3	3.4	0.16

**Table 2:** Estimated oral cancer mortality (all cases), both sexes, Europe (2022).

Both Sexes					Males				Females			
Cancer type	Number	ASR (World)	Crude rate	Cumulative risk	Number	ASR (World)	Crude rate	Cumulative risk	Number	ASR (World)	Crude rate	Cumulative risk
All cancers excl. non-melanoma skin cancer	1972982	105.8	263.9	11.4	1084483	134.5	300.2	14.3	888499	84.0	230.0	9.0
Lip, oral cavity	24253	1.6	3.2	0.19	17037	2.6	4.7	0.31	7216	0.69	1.9	0.08
Salivary glands	4303	0.23	0.58	0.02	2736	0.34	0.76	0.04	1567	0.13	0.41	0.01
Oropharynx	13027	0.90	1.7	0.11	10444	1.6	2.9	0.20	2583	0.30	0.67	0.04
Nasopharynx	2563	0.18	0.34	0.02	1889	0.29	0.52	0.03	674	0.09	0.17	0.01
Hypopharynx	9315	0.66	1.3	0.08	8226	1.3	2.3	0.16	1089	0.13	0.28	0.02
Oesophagus	47212	2.8	6.3	0.34	35963	4.9	10.0	0.60	11249	1.0	2.9	0.11

**Table 3:** Prevalence, both sexes, in 2022, Europe.

Both Sexes							Males						Females					
Cancer type	1-year	*Prop. (1-year)	3-year	*Prop. (3-year)	5-year	*Prop. (5-year)	1-year	*Prop. (1-year)	3-year	*Prop. (3-year)	5-year	*Prop. (5-year)	1-year	*Prop. (1-year)	3-year	*Prop. (3-year)	5-year	*Prop. (5-year)
All cancers excl. non-melanoma skin cancer	3206444	428.5	8315779	1111.4	12493752	1669.8	1644650	454.8	4190397	1158.9	6224690	1721.5	1561794	403.9	4125382	1067.0	6269062	1621.4
Lip, oral cavity	51714	6.9	134191	17.9	202275	27.0	34557	9.6	89398	24.7	134648	37.2	17157	4.4	44793	11.6	67627	17.5
Salivary glands	7901	1.1	20511	2.7	30860	4.1	4474	1.2	11328	3.1	16764	4.6	3427	0.89	9183	2.4	14096	3.7
Oropharynx	25306	3.4	65070	8.7	96199	12.9	19732	5.5	50831	14.1	75267	20.8	5574	1.4	14239	3.7	20932	5.4
Nasopharynx	3909	0.52	10338	1.4	15655	2.1	2838	0.78	7535	2.1	11340	3.1	1071	0.28	2803	0.72	4315	1.1
Hypopharynx	11841	1.6	25800	3.5	35602	4.8	10404	2.9	22508	6.2	31044	8.6	1437	0.37	3292	0.85	4558	1.2
Oesophagus	31665	4.2	57672	7.7	71996	9.6	24234	6.7	44122	12.2	54934	15.2	7431	1.9	13550	3.5	17062	4.4

*Proportions per 100 000.

Oral cancer remains a significant public health concern in Europe, with varying incidence and mortality rates across different cancer types.

#### 3.1.1 All cancers excluding non-melanoma skin cancer

The incidence of all cancers excluding non-melanoma skin cancer stands at a staggering 4,143,643 cases in Europe for 2022. Males account for approximately 52.4% of these cases, with an age-standardized rate (ASR) of 299.7 and a cumulative risk of 30.6%. Females, on the other hand, exhibit an ASR of 243.2 and a cumulative risk of 24.0%. In terms of mortality, all cancers excluding non-melanoma skin cancer contribute to 1,972,982 deaths in Europe. Males experience a higher mortality burden, with 1,084,483 deaths recorded, resulting in an ASR of 134.5 and a cumulative risk of 14.3%. Females show lower mortality rates, with 888,499 deaths, an ASR of 84.0, and a cumulative risk of 9.0%.

#### 3.1.2 Lip and oral cavity cancer

Lip and oral cavity cancer posed a considerable health challenge in Europe, with 62,103 incident cases reported in 2022. Among these cases, males accounted for a higher proportion (66.9%) compared to females (33.1%). The ASR for incidence was notably higher in males (6.4) than in females (2.2), indicating a significant gender disparity in the incidence of this cancer type. In terms of mortality, lip and oral cavity cancer led to 24,253 deaths, with males comprising 70.2% (17,037 deaths) and females 29.8% (7,216 deaths) of the total. The ASR for mortality was also higher in males (2.6) than in females (0.69). The cumulative risk of developing lip and oral cavity cancer by age 75 was 0.78 for males and 0.26 for females, highlighting a substantially higher risk for males. Prevalence rates per 100,000 individuals for 1-year, 3-year, and 5-year intervals were 6.9, 17.9, and 27.0 for males, and 4.4, 11.6, and 37.2 for females, respectively.

### 3.2 Etiology and risk factors

#### 3.2.1 Historical context

A historical review by Inchingolo et al. (13) examined how oral cancer was depicted throughout history. Their analysis of ancient Egyptian, Indian, Greek, and Roman texts suggests that these civilizations had a general understanding of tumors but could not distinguish between different types of cancerous and non-cancerous growths. Descriptions of oral malignancies from this era focus on destructive masses and non-specific surgical removal techniques. In addition, the study acknowledged the potential role of genetic factors in oral cancer and suggested that future research might explore cellular and molecular treatment approaches, alongside environmental factors.

#### 3.2.2 Epidemiological insights

A UK-based review by Conway et al. (14) investigated the distinct epidemiological profiles of oral squamous cell carcinoma. They identified tobacco and alcohol as primary risk factors, with a strong dose-response relationship. Socioeconomic status emerged as a concerning risk factor, with low education and income levels doubling the risk of OSCC. While dietary factors seem to have a limited influence, a high intake of fruits and vegetables offered protective effects (15). Notably, unlike many other cancers, obesity was not associated with increased risk of OSCC (16).

#### 3.2.3 Smokeless tobacco and regional variations

A meta-analysis conducted by Asthana et al. (17) investigated the association between smokeless tobacco (SLT) use and oral cancer risk. The study analyzed data from 37 studies across four WHO regions, identifying particularly high risks in Southeast Asia and the Eastern Mediterranean, especially for women users. Gutkha and pan masala were found to be especially harmful. The study emphasized the need for stricter regulations to control SLT use in these high-risk regions.

### 3.3 Emerging risk factors

Bugshan et al. (18) conducted a comprehensive review to investigate the etiology of OSCC, focusing on established and emerging risk factors. They identified cigarette smoking, alcohol consumption, smokeless tobacco (including shammah and shisha), and potentially malignant disorders (PMDs) as risk factors. These factors can increase the permeability of the oral epithelium, facilitating deeper penetration of carcinogens.

#### 3.3.1 HPV-related cancers

Fonseca et al. (19) conducted a systematic review to investigate the global prevalence of Human Papillomavirus (HPV) in OSCC. They found a significantly higher prevalence of HPV in OSCC, with a pooled prevalence of 10% in the oral cavity and 42% in the oropharynx. HPV16 was the most common genotype identified, particularly in oropharyngeal cancers. The prevalence of HPV-positive OSCC varied geographically, with higher rates in North America, Northern Europe, and Oceania. Further research is needed to confirm these trends and explore potential regional variations.

#### 3.3.2 Diagnosis

Early diagnosis of OSCC is crucial for improving patient outcomes. Despite advancements in treatment, many cases are diagnosed at advanced stages due to the insidious onset of the disease, limited public awareness, and challenges in early detection (20). The current standard for diagnosing oral cancer involves clinical examination and tissue biopsy, which, while effective, have limitations such as invasiveness, high costs, and potential sampling biases (21). This has driven a search for innovative diagnostic methods that are rapid, non-invasive, and cost-effective.

#### 3.3.3 Addressing delays

Lauritzen et al. (22) found that patient delays in seeking treatment for OSCC were associated with advanced-stage cancer only in Asian studies. Professional delays and total diagnostic delays did not generally correlate with advanced cancer. Time to treatment initiation (TTI) showed a correlation with overall survival in some studies, but not all. These findings suggest geographical variations in patient behavior and the need for improved public health education. The optimal timeframe for treatment initiation remains unclear and requires further investigation.

#### 3.3.4 Diagnostic intervals

Varela-Centelles et al. (23) conducted a quantitative systematic review to investigate the relative lengths of patient and primary care intervals in symptomatic oral cancer. They found that patient delays significantly contribute to the total time elapsed before diagnosis. Interventions focused on public awareness and health system optimization are crucial to address this issue.

#### 3.3.5 Treatment

The treatment for OSCC is individualized based on several factors, including the stage of the cancer, tumor location, patient health status, and personal preferences. The primary objective of treatment is to eliminate the cancer, preserve function and appearance, and minimize the risk of recurrence. Treatment strategies typically involve a multidisciplinary approach, integrating surgery, radiation therapy, and chemotherapy, depending on the cancer’s staging and characteristics.

##### 3.3.5.1 Stage 0 (carcinoma in situ)

Carcinoma *in situ* is an early stage of OSCC confined to the surface layer of the oral epithelium. The primary treatment involves surgical excision to remove the cancerous lesion and a small margin of healthy tissue. Procedures such as Mohs surgery, surgical stripping, or thin resection may be employed (24). Radiation therapy may be considered if the cancer recurs or is not fully removed by surgery. Lifelong follow-up is essential to monitor for recurrence or new cancers, especially in patients who continue to smoke, as smoking increases the risk of developing additional malignancies (25).

##### 3.3.5.2 Stages I and II

These are typically treated with surgery to remove the tumor. For smaller tumors, surgery alone is often sufficient (7). However, for cancers involving the lip or front of the tongue, lymph node dissection may be performed if there is a risk of nodal spread.

#### 3.3.6 Submandibular gland preservation

Iocca et al. (26) investigated the involvement and preservation of the submandibular gland (SMG) during surgery for OSCC. The study suggests that SMG preservation can be considered for select patients with early-stage cancer to maintain salivary function, provided there is no involvement of the floor of the mouth or level Ib lymph nodes.

##### 3.3.6.1 Stages III and IVA

Locally advanced oral cavity cancers require a combination of treatments to effectively manage the disease. Surgery is typically the first-line treatment to remove the primary tumor and any involved lymph nodes. This is often followed by adjuvant radiation therapy or chemoradiation to reduce the risk of recurrence (27).

#### 3.3.7 Hyperfractionated accelerated radiotherapy (HART)

Sakso et al. (28) investigated the use of HART with nimorazole for patients with head and neck squamous cell carcinoma. This approach is suitable for patients who are unable to undergo surgery or for those with locally advanced cancers that are still potentially removable. The treatment demonstrated a favorable 3-year loco-regional failure rate and overall survival. Long-term side effects seem comparable to existing chemo-radiation treatments. Future research will explore combining HART with concurrent chemotherapy for potentially superior outcomes.

##### 3.3.7.1 Stages IVB and IVC

Advanced-stage OSCCs have typically spread to surrounding tissues or distant organs. For Stage IVB cancers that are not surgically removable, treatment options may include radiation therapy alone, chemoradiation, or chemotherapy to manage symptoms and control disease progression. Stage IVC cancers, which involve distant metastases, are generally treated with systemic therapies such as targeted therapy, or immunotherapy. The primary goal in these cases is palliative care, aiming to improve quality of life and extend survival.

#### 3.3.8 Immunotherapy

Siu et al. (29) conducted a clinical trial (CONDOR) which investigated the efficacy and safety of durvalumab (anti-PD-L1) with or without tremelimumab (anti-CTLA-4) for metastatic head and neck squamous cell carcinoma with low or no PD-L1 expression. Both durvalumab and the combination with tremelimumab showed clinically meaningful improvement in overall survival, highlighting its potential to improve survival outcomes in patients with advanced disease.

#### 3.3.9 Targeted therapy

Gebre-Medhin et al. (30) evaluated the effectiveness of cisplatin versus cetuximab for locoregionally advanced HNSCC. The study was stopped early due to insufficient enrollment. While overall survival did not improve with cetuximab, locoregional control was worse. These findings suggest cisplatin remains the standard treatment for both HPV-positive and HPV-negative HNSCC, until further studies identify potential subgroups that might benefit from cetuximab.

#### 3.3.10 Recurrent oral squamous cell carcinoma

For recurrent OSCCs, treatment options depend on the extent of recurrence, prior treatments, and the patient’s overall health. Surgical resection may be considered if the recurrence is localized and resectable. Radiation therapy, chemotherapy, or a combination of these may be used for more advanced recurrences. Targeted therapies and immunotherapy are also being investigated for their potential to improve outcomes in recurrent OSCCs.

#### 3.3.11 Personalized medicine

R. Galot et al. (31) proposed a promising approach for recurrent OSCC with the EORTC-1559-HNCG biomarker-driven umbrella trial. This study highlights the potential benefits of personalized medicine in treating oral cavity cancer. By matching patients with appropriate treatments based on their tumor biology, the trial seeks to improve treatment outcomes. While DNA-level biomarkers may have limitations, further research is needed to identify additional biomarkers and develop more effective targeted therapies.

#### 3.3.12 Prognosis

The EUROCARE-5 study (32), a pan-European initiative investigating cancer survival and care, aimed to modernize cancer survival monitoring by analyzing data for patients diagnosed through 2007 and followed up to December 31st, 2008 (33). This analysis provided valuable insights into the survival and prognosis of individuals diagnosed with OSCC and pharyngeal cancers. Examining data across different age groups and genders, the study revealed significant variations in life expectancy at the time of diagnosis as shown in **Table 4**. For individuals diagnosed between the ages of 15 to 44, males with OSCC and pharynx cancers had a life expectancy of 1.7 years, while females had a slightly higher expectancy of 2.4 years. Similarly, for esophageal cancer, males in the same age group had a life expectancy of 0.7 years, while females had a slightly lower expectancy of 0.8 years. As age increased, life expectancy generally decreased across all cancer types. For instance, males and females aged 65 to 74 with oral cavity and pharynx cancers had a life expectancy of 1.5 and 2.2 years, respectively, while those with esophageal cancer had a life expectancy of 0.5 and 0.6 years, respectively. The study highlights the need for individualized treatment approaches for OSCC, given the diverse prognosis associated with age, gender, and cancer type.

**Table 4:** Life expectancy of fatal cases (years) at diagnosis by cancer type, sex and age in Europe.

Age at diagnosis (years)								
Cancer type	15-44		45-54		55-64		65-74	
	M	F	M	F	M	F	M	F
All types	1.2	2.7	1.0	2.3	1.0	1.6	1.0	1.0
Oral cavity and pharynx	1.7	2.4	1.8	2.7	1.8	2.4	1.5	2.2
Oesophagus	0.7	0.8	0.7	0.7	0.6	0.7	0.5	0.6

Beyond life expectancy at diagnosis, an important consideration in oral cancer prognosis is the time to cure. **Table 5** presents a comprehensive analysis of “time to cure” for various cancer types in Europe. This metric signifies the estimated number of years needed for patients to achieve a favorable outcome – a 5-year conditional relative survival (5-year CRS) exceeding 95%. The age group 15-44 years demonstrated the most rapid progression towards achieving a favorable 5-year conditional relative survival (5-year CRS) exceeding 95%. Males within this cohort required an average of 6 years, while females exhibited a slightly longer duration of 8 years. This trend of increasing “time to cure” with age continued for the 45-54 age group, where both sexes displayed a similar duration of 9 years. Individuals aged 55-64 displayed a further increase, necessitating 10 years for both males and females. This pattern persisted in the oldest age group (65-74), with males requiring a median of 13 years and females requiring a median of 12 years to reach the desired 5-year CRS benchmark. Focusing on OSCC and pharyngeal cancers specifically, the data unveils a nuanced temporal pattern. While a similar age-related trend exists, the time to cure is generally longer compared to all cancers. Males diagnosed between 15-44 years old needed 8 years on average, while females in the same age group required 7 years. Interestingly, this sex disparity narrowed or even reversed in older age cohorts. For example, the 45-54 age bracket exhibited a reversal, with males requiring 12 years and females needing 13 years. Notably, individuals aged 55-64 displayed a convergence, with both sexes requiring 15 years. Finally, the 65-74 cohort exhibited a divergence again, with males needing 17 years and females requiring 18 years.

**Table 5:** Time to cure measured as years to reach 5-year conditional relative survival (5-year CRS) >95% by cancer type, sex and age in Europe.

Age at diagnosis (years)								
Cancer type	15-44		45-54		55-64		65-74	
	M	F	M	F	M	F	M	F
All types	6	8	9	9	10	10	13	12
Oral cavity and pharynx	8	7	12	13	15	15	17	18
Oesophagus	6	6	6	6	7	6	7	6

While the reasons behind the initial gap favoring females and the later reversal in older age groups are not entirely clear, it highlights potential biological or behavioral factors influencing treatment outcomes. Achieving long-term improvements in prognosis necessitates a deeper understanding of the underlying biological processes driving tumor initiation and progression.

#### 3.3.13 Oral epithelial dysplasia (OED)

Nevanpaa et al. (34) conducted a retrospective registry-based study in Southwest Finland to investigate the malignant transformation rate of oral epithelial dysplasia (OED) to oral squamous cell carcinoma (OSCC) (**Table 6**). They found that 10.9% of OED patients developed OSCC during a mean follow-up of 5.5 years. OED patients had a significantly increased risk (44.7-fold) of developing OSCC compared to the general population.

**Table 6:** Transformation of oral epithelial dysplasia (OED) to oral squamous cell carcinoma (OSCC).

OED grade (n)	OSCC cases	Transformation rate
Mild (390)	32	8.1
Moderate (143)	23	16.1
Severe (13)	5	38.5

#### 3.3.14 HPV infection

Christianto et al. (35) conducted a systematic review and meta-analysis to investigate the impact of HPV infection on prognosis in oral squamous cell carcinoma (OSCC). Surprisingly, HPV positivity was associated with worse overall survival (OS) and distant control (DC) compared to HPV-negative OSCC. This study highlights the need to re-evaluate the prognostic role of HPV infection in OSCC.

#### 3.3.15 p53 gene mutations

Ragos et al. (36) investigated the critical role of p53 gene deregulation, particularly its mutation status, in oral squamous cell carcinoma (OSCC). Mutations in the p53 gene, affecting roughly 70% of cases, can promote cancer cell growth, invasion, and resistance to treatment. Identifying specific p53 mutations may help guide treatment decisions and prognosis. Further research is needed to understand the relationship between HPV infection and p53 alterations in OSCC.

## 4 Discussion

### 4.1 Key trends and knowledge gaps in European OCC

This review examined trends in oral squamous cell carcinoma (OSCC) across Europe, leveraging data from GLOBOCAN 2022 alongside the EUROCARE-5 study. A significant finding is the marked geographic disparity in disease burden, with Eastern and Central Europe exhibiting notably higher incidence and mortality rates compared to Northern and Western Europe. These regional differences are likely influenced by a combination of risk factors, including tobacco and alcohol use, socioeconomic disparities, occupational exposures (e.g., asbestos) (5, 37) and dietary deficiencies (38). Countries like Hungary and Russia face higher OSCC burdens (39). This disparity may indicate that established risk factors like tobacco use and alcohol consumption, could be more prevalent in these regions (40). In contrast, public health interventions in countries like Ireland and France have led to reductions in OSCC incidence, although disparities in outcomes persist. Future research should investigate the specific risk factor profiles of diverse European populations with a focus on modifiable behaviors and potential environmental exposures. This knowledge can guide targeted prevention efforts, potentially including culturally sensitive public health campaigns and resource allocation for smoking cessation programs and alcohol abuse interventions. Addressing the knowledge gaps through focused research and targeted interventions can help reduce the burden of OCC and improve outcomes across Europe.

### 4.2 Gender disparity in OSCC burden

OSCC exhibits a persistent gender disparity, with males consistently experiencing a higher burden of the disease. This disparity is likely influenced by a complex interplay of biological, behavioral, and social factors. Males may be more susceptible to developing OCC due to differences in tumor biology and hormonal influences, and they generally exhibit higher rates of risky behaviors such as smoking and alcohol consumption. Additionally, males tend to have lower health-seeking behaviors and less social support networks, often leading to delayed diagnosis and treatment (7). Socioeconomic factors, including race and income, can exacerbate gender disparities in OSCC outcomes (41). Sex-based differences in HPV prevalence and immune response could also play a role, as HPV-positive oropharyngeal cancers are more common in males (42). The EUROCARE-5 study underscores this gender disparity, revealing significantly higher mortality rates among men compared to women. This suggests that the gender gap in OSCC outcomes is not solely due to biological factors but also involves behavioral, social, and healthcare-related factors (32). Research into these factors could explore hormonal influences, healthcare access patterns, and social norms related to tobacco and alcohol use (**Table 7**). Additionally, investigations into potential sex-based differences in HPV prevalence and immune response could be informative (43, 44). These findings can inform the development of gender-sensitive prevention and treatment strategies, such as tailored smoking cessation programs for men and targeted HPV vaccination campaigns for young women.

**Table 7:** Characteristics of included studies.

Author(s)	DOI	Country/Region	Type of Cancer Studied	Study Design	Number of Cases/Studies included	Year of Publication
Inchingolo et al. (13)	https://doi.org/10.3390/ijerph17093168	Egypt, India, Greece and Rome	Oral Cancer	Historical Review	145 studies	2020
Conway et al. (14)	https://doi.org/10.1038/sj.bdj.2018.922	UK (England, Wales, Scotland, and Northern Ireland)	Oral Cavity Cancer	Systematic Review	35 studies	2018
Asthana et al. (17)	https://doi.org/10.1093/ntr/nty074	Southeast Asia, Eastern Mediterranean, Europe, Americas	Oral Cancer	Meta-Analysis	37 studies	2019
Bugshan et al. (18)	https://doi.org/10.12688/f1000research.22941.1	Global	Oral Squamous Cell Carcinoma (OSCC)	Review	77 studies	2020
Fonseca et al. (19)	https://doi.org/10.1007/s00784-023-05425-0	North America, Northern Europe, Oceania, Asia, South America, Africa	HPV-related OSCC	Systematic Review	65 studies	2023
Lauritzen et al. (22)	https://doi.org/10.1080/0284186X.2021.1931712	Australia, Asia, Europe, North and South America	Oral Cavity Cancer	Systematic Review	16 studies	2021
Varela-Centelles et al. (23)	https://doi.org/10.1111/coa.12919	Europe, USA, India, Australia, Japan, Argentina and Iran	Oral Cancer	Systematic Review	22 studies	2018
Iocca et al. (26)	https://doi.org/10.1007/s00405-023-08007-8	Europe	Oral Cavity Carcinoma (OCC)	Meta-Analysis	24 studies	2023
R. Galot et al. (31)	https://doi.org/10.1093/annonc/mdy452	Belgium, France, Italy, and UK	Recurrent/Metastatic SCCHN	Review	Multiple trials on biomarker-driven strategies for SCCHN	2018
Sakso et al. (28)	https://doi.org/10.1080/0284186X.2019.1658897	Denmark	Locally Advanced HNSCC	Clinical Trial	50 patients	2020
Siu et al. (29)	10.1001/jamaoncol.2018.4628	North America, Europe, and Asia Pacific	Recurrent/Metastatic HNSCC	Clinical Trial	267 patients	2019
Gebre-Medhin et al. (30)	https://doi.org/10.1200/JCO.20.02072	Sweden	Locoregionally Advanced HNSCC	Clinical Trial	298 patients	2021
Nevanpaa et al. (34)	https://doi.org/10.1038/s41598-022-12441-9	Southwest Finland	Oral Epithelial Dysplasia (OED) and OSCC	Meta-Analysis	571 patients	2022
Christianto et al. (35)	https://doi.org/10.1002/lary.29996	Global	HPV-related OSCC	Systematic Review	22 studies	2021
Ragos et al. (36)	JBUON 2018; 23(6): 1572	Global	Oral Cavity Cancer	Review	Extensive genomic analyses	2021

### 4.3 Impact of age on OSCC prognosis

The EUROCARE-5 study also highlights the significant impact of age on the prognosis of oral squamous cell carcinoma (OSCC). While younger patients tend to have a longer life expectancy compared to older patients, studies suggest that OSCC in younger individuals can exhibit a more aggressive phenotype, characterized by genetic alterations, lymphatic metastasis, and poorly differentiated tumors (45). This may lead to poorer outcomes despite the potential for longer survival. Older patients with OSCC often face additional challenges due to comorbid conditions, which can complicate treatment and lead to poorer outcomes (46). Moreover, the concept of “time to cure” underscores the potentially longer and more complex treatment journey for older adults (32). These factors necessitate age-specific treatment optimization strategies. Future research should focus on tailoring therapies to the unique needs of each age group. For younger patients, this may involve exploring the tolerability of aggressive treatment regimens, while for older patients, identifying treatment options with fewer side effects is crucial. Additionally, developing supportive care strategies tailored to the specific challenges faced by both younger and older OSCC patients is essential.

### 4.4 A focus on emerging risk factors

The etiological landscape for OSCC is evolving, with the emergence of human papillomavirus (HPV), particularly genotypes 16 and 18, as a significant risk factor for oropharyngeal cancer (41). While traditional risk factors like tobacco and alcohol remain crucial, HPV’s role in OSCC has garnered increasing attention. Studies suggest that HPV-positive cases may exhibit distinct biological behaviors and potentially better prognoses compared to HPV-negative cases (47). Furthermore, HPV may interact with the oral microbiome, contributing to the carcinogenic process. Future research should prioritize elucidating the interplay between established and emerging risk factors, including HPV infection, genetic susceptibility, and the influence of the microbiome (48). This knowledge can inform the development of more effective preventive strategies, such as HPV vaccination programs alongside continued efforts to reduce tobacco use and alcohol consumption (49).

### 4.5 Optimizing diagnosis and treatment for improved outcomes

Minimizing diagnostic delays through public awareness campaigns aimed at early symptom recognition and improved healthcare system efficiency is crucial for earlier diagnoses and improved survival rates. Additionally, research into patient navigation programs and the role of telemedicine in facilitating timely access to specialists could be beneficial. The promise of precision medicine in OSCC management holds immense potential (50). Continued exploration of targeted therapies based on specific tumor mutations and advancements in combined radioimmunotherapy approaches offer significant promise for improved patient outcomes, particularly for those with advanced or recurrent disease (51). Additionally, research into the potential of minimally invasive surgical techniques and personalized rehabilitation programs tailored to improve patient quality of life after treatment could be valuable areas of exploration.

## 5 Strengths and limitations

The systematic review provides a comprehensive overview of Oral Squamous Cell Carcinoma in Europe, encompassing a wide range of topics and incorporating the latest data available from the GLOBOCAN 2022 database. This ensures that the findings are current and relevant to the current understanding of the disease burden and trends in Europe. The review highlights emerging risk factors such as HPV infection and smokeless tobacco use, providing invaluable insights into the disease’s impact within the European context. It benefits from a multidisciplinary approach, integrating findings from epidemiology, oncology, genetics, and public health to offer a comprehensive understanding of the disease’s etiology, risk factors, treatment options and prognosis. It also identifies significant research gaps, guiding future studies and prioritizing research efforts. By providing an up-to-date overview of OSCC in Europe, the review offers a valuable resource for researchers, clinicians, and policymakers working on this important public health issue. Limitations to this article include publication bias, language bias, and limited data on long-term outcomes which may hinder a comprehensive understanding of the disease’s impact. Heterogeneity of studies in design, population, and methodology can make it challenging to draw consistent conclusions. Underrepresentation of certain subpopulations may limit the generalizability of the findings and the assessment of study quality.

## 6 Conclusion

This multi-source review examined the contemporary epidemiology of OSCC in Europe, identifying concerning trends and knowledge gaps. Geospatial disparities in disease burden necessitate targeted interventions tailored to regional risk profiles. The persistent sex disparity calls for further investigation into biological, behavioral, and social determinants of health to inform sex-specific strategies. The EUROCARE-5 study highlighted the influence of age on prognosis, emphasizing the need for age-optimized treatments. Future research should prioritize elucidating the interplay between risk factors, including HPV infection, in OSCC development. Additionally, minimizing diagnostic delays and advancements in personalized medicine with targeted therapies and radioimmunotherapy hold promise for improved outcomes. By addressing these knowledge gaps and implementing research recommendations, Europe can strive for a future with a diminished OSCC burden and demonstrably better patient outcomes.

## Data Availability

The datasets presented in this study can be found in online repositories. The names of the repository/repositories and accession number(s) can be found in the article/supplementary material.
